# Adverse childhood experiences influence markers of neurodegeneration risk in older adults

**DOI:** 10.1002/alz.70523

**Published:** 2025-07-30

**Authors:** Deborah K. Rose, Gabrielle Pfund, Jillian K. Lee, Andy J. Liu, Andreana Benitez, Negar Fani, Keenan A. Walker, Michelle M. Mielke, James R. Bateman

**Affiliations:** ^1^ Department of Psychiatry Johns Hopkins University School of Medicine Baltimore Maryland USA; ^2^ Department of Human Development & Family Science Auburn University Auburn Alabama USA; ^3^ Department of Epidemiology & Prevention Division of Public Health Sciences Wake Forest University School of Medicine Winston‐Salem North Carolina USA; ^4^ Department of Neurology Duke University School of Medicine Durham North Carolina USA; ^5^ Department of Neurology Medical University of South Carolina Charleston South Carolina USA; ^6^ Department of Psychiatry & Behavioral Sciences Emory University School of Medicine Atlanta Georgia USA; ^7^ Intramural Research Program National Institute on Aging Baltimore Maryland USA; ^8^ Department of Neurology Wake Forest University School of Medicine Winston‐Salem North Carolina USA

**Keywords:** adverse childhood experiences, dementia risk, neurodegeneration biomarkers, neuroimaging signatures, white matter integrity

## Abstract

**INTRODUCTION:**

Adverse childhood experiences (ACEs) disrupt brain development and increase vulnerability to Alzheimer's disease and related dementias (ADRD). We explored how ACEs impact neuroimaging, plasma biomarkers, and cognition in older adults.

**METHODS:**

Data from 214 participants aged ≥ 55 years were analyzed using linear and logistic regression, adjusting for demographic covariates.

**RESULTS:**

Financial need associated negatively with Montreal Cognitive Assessment scores (β = –0.10, *p *= 0.011). Lower mean diffusivity across white matter tracts associated with parental violence (β = –0.01, *p *= 0.03). Lower glial fibrillary acidic protein associated with parental intimidation (β = –0.07, *p *= 0.01) and parental violence (β = –0.18, *p* = 0.006). Family problems and separation (β = –0.16, *p *= 0.003), financial need (β = –0.1, *p *= 0.04), and parental intimidation (β = –0.05, *p *= 0.01) inversely associated with neurofilament light chain.

**DISCUSSION:**

Findings challenge the notion that ACEs uniformly accelerate neurodegeneration. Longitudinal studies are needed to determine whether these results reflect resilience, survivorship, or cohort‐specific factors influencing ADRD risk.

**Highlights:**

- Adverse childhood experiences (ACEs) may elicit compensatory neural responses in aging.
- Financial need was associated with lower global cognition (Montreal Cognitive Assessment scores).
- Some ACEs (e.g., financial need, parental intimidation) linked to lower plasma neurofilament light chain.
- Parental violence linked to lower glial fibrillary acidic protein and mean diffusivity values, implying intact white mattery integrity.

## 1 BACKGROUND

Childhood adversity, such as abuse, neglect, and family instability, is a pervasive public health issue affecting millions in the United States.^1^ These adverse childhood experiences (ACEs) have far‐reaching implications, extending beyond individual health to societal costs, reduced productivity, and generational impacts.^2^, ^3^ ACEs can disrupt normal developmental trajectories, with lasting effects on brain development, physiological function, and adult health outcomes.^4^, ^5^ ACEs can become biologically embedded, influencing brain function and physiology.^6^, ^7^ Thus, these experiences can set the stage for a lifetime of increased vulnerability to various chronic conditions, including cardiometabolic diseases^8^, ^9^ and cognitive decline.^10^, ^11^, ^12^, ^13^ This broad and multifaceted impact underlines the importance of understanding the mechanisms through which ACEs influence long‐term health.

The relationship between ACEs and Alzheimer's disease and related dementias (ADRD) has gained increasing attention in light of the growing prevalence of ADRD, especially in minoritized populations most at risk of ADRD.^14^, ^15^ Although ACEs are linked to an elevated risk of cognitive impairment and dementia,^10^, ^11^, ^12^, ^13^ the pathways that connect early adversity to these late‐life outcomes remain poorly understood. These pathways may include chronic activation of the hypothalamic–pituitary–adrenal (HPA) axis,^16^, ^17^ resulting in inflammatory and neurodegenerative processes that compromise brain health.^18^ In addition, chronic activation of the sympathetic nervous system in response to early adversity drives transcriptional changes, including the conserved transcriptional response to adversity, which influences immune function and inflammation.^19^ Studies also have highlighted structural and functional changes in the brain associated with ACEs,^20^ such as reduced cortical surface area in regions critical for cognition, including the prefrontal cortex and temporal lobes.^21^ Notably, ACEs are associated with altered white matter integrity, including reduced fractional anisotropy (FA) in the corpus callosum and other major white matter tracts, indicating compromised structural connectivity.^22^, ^23^ Integrating plasma and neuroimaging biomarkers offers a promising avenue to uncover these mechanisms and advance our understanding of how ACEs contribute to ADRD risk.

Despite these advances, significant gaps remain in the literature. Most research on ACEs and brain health focuses on younger populations or single factors, such as cognition or inflammation, rather than integrating multiple factors in older adults.^24^ Further, the majority of studies have under‐sampled racially and ethnically diverse groups.^25^ For instance, the Alzheimer's Disease Neuroimaging Initiative (ADNI) has historically been overwhelmingly White, with > 90% of participants identifying as White in both ADNI‐2 and ADNI‐3 cohorts.^26^ It is important to note that while certain early‐life adversities may occur more frequently in some racial and ethnic groups due to structural and social inequities,^27^, ^28^ the conventional ACEs can occur in any racial or ethnic group and are not confined to a single population.^4^ Nevertheless, understanding the interplay among ACEs, cognition, and fluid or neuroimaging biomarkers in diverse populations is essential to address health disparities and tailor interventions to mitigate ADRD risk.

To address these gaps, this study leverages data from the Wake Forest Alzheimer's Disease Research Center (WF ADRC) community cohort to examine direct associations of ACEs with cognition, plasma ADRD biomarkers (glial fibrillary acidic protein [GFAP], neurofilament light chain [NfL], amyloid beta 42/40 [Aβ42/40] ratio, and phosphorylated tau [p‐tau] 181), and cerebrovascular neuroimaging measures (white matter hyperintensity [WMH], diffusion tensor imaging metrics, and neurite orientation dispersion and density imaging [NODDI] indices).

## 2 METHODS

### 2.1 Study design and participants

All participants were enrolled in the Alzheimer's Disease Clinical Core cohort of the WF ADRC and underwent standardized evaluations in accordance with the National Alzheimer's Coordinating Center protocol, which meets Uniform Data Set requirements. Recruitment was community based, including advertisements and outreach to underrepresented communities. Specific inclusion and exclusion criteria for the Clinical Core are described elsewhere.^29^ The Adverse Childhood Experiences Questionnaire (ACE‐Q) was administered either through the mail or in person during the study visit in April 2022 through July 2023. Our study integrated clinical, neuroimaging, and plasma biomarker data with detailed assessments of ACEs from the WF ADRC. The analytic sample consisted of 214 participants ≥ 55 years old who had complete demographic data (age, sex, race, and education), available adjudicated clinical diagnosis of normal cognition (NC) or mild cognitive impairment (MCI), and a completed ACE‐Q. Participants with missing data on ACE measures (*n* = 13), neuroimaging (*n* = 106), or biomarker variables were excluded (*n* = 56). Two participants who self‐identified as Asian were also excluded from stratified analyses due to small sample size. There were no participants identifying as Hispanic or Latino in this analytic sample. Ethical approval for the study was obtained from the institutional review board at Wake Forest University (IRB00025540), and all participants provided informed consent before study participation.

### 2.2 ACEs

ACEs were assessed using the ACE‐Q (adopted from the Childhood Trauma Questionnaire),^30^ which includes items evaluating five ACE domains: emotional neglect, family problems and separation, financial hardship, parental intimidation, and parental violence.^31^ Participants responded to 14 items on a scale from 1 (never) to 5 (always) across all domains except family problems and separation, which was dichotomized (yes = 1, no = 2). Composite scores were calculated for each ACE domain by summing individual item responses. Each ACE subscale score was computed as the sum of individual item responses. A higher total score reflected more significant overall childhood adversity. Scores were treated as continuous variables to capture the full range of experiences, and participants were categorized as having experienced an ACE if they reported any level of exposure. To examine potential demographic differences, two‐sample *t* tests were conducted to compare ACE scores by sex and by race.

*Emotional neglect* was scored based on six items (score range: 0–25; e.g., “When you were growing up, did you feel loved?”). *Family problems and separation* were assessed through eight items (score range: 0–12) measuring family disruptions (e.g., “Did your parents ever separate or divorce?”). *Financial need* was evaluated by two items (score range: 0–10) assessing the frequency of experiences such as food insecurity and inadequate clothing (e.g., “When you were growing up, how often was there not enough to eat?”). *Parental intimidation* included four items (score range: 0–20) assessing the extent of verbal and physical intimidation by parents (e.g., “How often did an adult in your home say mean or hurtful things to you?”). *Parental violence* assessed two items (score range: 0–10) related to physical punishment and domestic violence (e.g., “How often were you punished with a belt or other hard object?”).

**RESEARCH IN CONTEXT**

1. **Systematic review**: The authors conducted a literature review using PubMed and relevant conference abstracts to evaluate prior studies linking adverse childhood experiences (ACEs) to biomarkers of Alzheimer's disease and related dementias (ADRD). Most existing research emphasizes the detrimental effects of ACEs on brain health; however, few studies have explored domain‐specific ACEs in relation to plasma biomarkers and neuroimaging in diverse older populations.
2. **Interpretation**: This study revealed unexpected associations between specific ACEs and lower levels of neurodegeneration biomarkers (glial fibrillary acidic protein, neurofilament light chain) and lower mean diffusivity neuroimaging values. These findings suggest that ACEs may influence neurobiological aging in complex or compensatory ways, challenging assumptions that ACEs uniformly accelerate neurodegeneration.
3. **Future directions**: Longitudinal studies are needed to examine how ACEs affect neurodegeneration trajectories over time. Future work should incorporate additional neuroimaging metrics, inflammatory biomarkers, and stratification by genetic risk (e.g., apolipoprotein E ε4) to clarify mechanisms of resilience or vulnerability among individuals with early‐life adversity.

### 2.3 Neuroimaging

Neuroimaging data were collected using a research‐dedicated 3‐Tesla Siemens Skyra magnetic resonance imaging (MRI) scanner equipped with a 32‐channel head coil. Imaging sequences included T1‐weighted, T2 fluid‐attenuated inversion recovery (FLAIR), and diffusion tensor imaging (DTI). Structural MRI was used to derive total intracranial volume and WMH volume, which were quantified via T2/FLAIR sequences. DTI provided measures of FA and mean diffusivity (MD), which serve as markers of white matter microstructure. FA reflects the directionality of water diffusion and is typically interpreted as a measure of fiber organization and myelination, while MD quantifies the overall magnitude of water diffusion, with higher values generally indicating reduced white matter integrity due to axonal degeneration, demyelination, or increased extracellular water content.^32^ All neuroimaging metrics were calculated using the Johns Hopkins University (JHU) atlas for standardized analysis. Detailed image acquisition parameters are provided in the Supplementary Methods in supporting information.

WMH volume representing lesions of presumed ischemic origin was segmented using the lesion growth algorithm implemented in the Lesion Segmentation Tool toolbox v2.0.15 within SPM12, using FLAIR and T1 images. WMH volume masks were manually edited by trained observers as necessary. Details on DTI processing are provided in the supporting information. Briefly, the JHU DTI atlas was applied to template‐space FA and free water images to derive mean signals across all supratentorial white matter tracts.

### 2.4 Adjudicated clinical cognitive diagnosis and global cognition (Montreal Cognitive Assessment scores)

Cognitive diagnoses were determined through expert panel consensus, which involved comprehensive review of clinical, neuroimaging, and cognitive data. This process followed current National Institute on Aging–Alzheimer's Association criteria for diagnosing MCI, ADRD, and their subtypes.^33^, ^34^ The panel included experienced investigators (e.g., neuropsychologists, neurologists, geriatricians) who specialize in evaluating cognitive function and identifying impairment in older adults. In addition to adjudicated diagnostic categories, we examined global cognitive performance using total scores from the Montreal Cognitive Assessment (MoCA).

The MoCA is a widely used tool to screen multiple cognitive domains, including memory, attention, visuospatial abilities, language, and executive function.^35^ It is particularly sensitive to detect MCI, making it a valuable instrument in populations at risk for ADRD.^35^ The assessment includes tasks such as word recall, clock drawing, and serial subtraction, yielding a total score ranging from 0 to 30, with higher scores indicating better cognitive performance. Adjudication of syndromic cognitive diagnosis occurred yearly based on clinical and cognitive assessments and was independent of neuroimaging and fluid biomarkers. Only participants with NC or MCI were included in this study. MCI was based on clinical criteria established by National Alzheimer's Coordinating Center guidelines.^36^

### 2.5 ADRD and neurodegeneration plasma biomarkers

Plasma biomarkers (GFAP, NfL, p‐tau181, and Aβ42/40 ratio) were measured using validated immunoassay techniques. Plasma NfL, GFAP, Aβ42, and Aβ40 (*n* = 209) were analyzed in duplicate using the single‐molecule array (Simoa) Neurology 4‐Plex E assay, and p‐tau181 was measured using the Simoa pT181 Advantage version 2 kits on a Simoa HD‐X system^37^ (Quanterix Corp.).^36^, ^37^ Immunoassay methods were based on Simoa technology, which provides high sensitivity and specificity for detecting these proteins in peripheral blood. Plasma p‐tau 217, another biomarker that reflects early amyloid plaque pathology^38^ and has high specificity and sensitivity for AD diagnosis,^39^ was not available in this cohort at the time of analysis.

Visual inspection and diagnostic analyses using Cook distance (threshold = 4/n)^40^ assessed biomarker data for influential outliers. One GFAP data point (678 pg/mL) was flagged during initial inspection, but this was not influential. For NfL, two influential observations (> 40 pg/mL) exceeded the threshold and were excluded. To facilitate interpretation and comparability, all biomarker values were *Z* score standardized, such that each value represents the number of standard deviations from the sample mean.^38^, ^39^, ^40^, ^41^ We also conducted exploratory race‐stratified visualizations to illustrate trends in ACE–biomarker associations.

### 2.6 Covariates

Covariates included age, sex, race, education, and medical comorbidities collected during participant evaluations. Age was recorded in years at the time of data collection and was mean‐centered in regression models, meaning a 1 unit change reflects a 1 year difference from the cohort mean age. Sex was reported as male or female (coded as 1 = male, 2 = female). Race was self‐reported and categorized as Black or African American (2), and White (3). Race was included as a covariate to account for potential variation across racial subgroups in social and environmental exposures that may influence outcomes of cognition and biomarkers of plasma and neuroimaging. Education was measured as total years of schooling completed and was mean centered; thus, a 1 unit increase represented one additional year of education relative to the cohort mean. Medical comorbidities were assessed through participant self‐report and medical history, including presence or absence of hypertension, sleep apnea, diabetes mellitus, traumatic brain injury, and cardiovascular disease. These comorbidities were dichotomously coded (0 = absent, 1 = present). All covariates were included in regression models to adjust for potential confounding effects on the relationships between ACEs and cognitive or biomarker outcomes

### 2.7 Statistical analyses

All analyses were performed using *R* version 4.4.1. Demographic characteristics (e.g., age, sex, race, educational attainment), as well as associations between ACEs and adjudicated clinical diagnoses (NC, MCI), and ACEs and total MoCA scores (global cognition) were examined in a subset of 214 participants. A Mann–Whitney *U* test was additionally conducted to ascertain differences in mean ACE scores between clinical diagnosis groups, and Spearman correlation to evaluate differences in ACE scores by cognitive performance on the MoCA. Simple linear regression models assessed the relationships between ACEs separately and neuroimaging measures; these models included 149 participants for FA, 140 for MD, and 147 for NODDI. Mean values for each metric were computed across all white matter regions of interest defined by the JHU white matter atlas to account for inter‐individual variation in regional tract size. In addition, simple linear regression models assessed associations between ACEs and plasma biomarkers (p‐tau181, NfL, GFAP, Aβ42/40 ratio) using data from 182 participants. All models were adjusted for age, sex, race, educational attainment, and medical comorbidities of hypertension, diabetes mellitus, sleep apnea, traumatic brain injury, and cardiovascular disease. Statistical significance was defined as *p* < 0.05.

## 3 RESULTS

### 3.1 Demographics and clinical characteristics

This study analyzed data from a subset of participants (*n* = 214) in the WF ADRC cohort. The mean age of participants was 68.8 years (standard deviation [SD] = 7.91; Table 1). The cohort was predominantly female (65.4%, *n* = 140). Racial composition was 83.6% White (*n* = 179) and 16.4% Black or African American (*n* = 35). Educational attainment was high, with participants reporting a mean of 16.1 years of education (SD = 2.43). The mean years of educational attainment was 15.7 among Black participants and 16.2 among White participants. Of the 214 participants, 146 (68.2%) were diagnosed with NC, and 68 (31.8%) with MCI (Table 1).

**TABLE 1:** Demographic and clinical characteristics of the Wake Forest Alzheimer's Disease Research Center cohort.

Characteristic	Analytic sample (*N* = 214)
Demographics	*M (SD)*
Age in years	68.8 (7.91)
Years of education	16.1 (2.43)

	No. (%)
Sex	
Male	74 (34.6)
Female	140 (65.4)
Race	
White	179 (83.6)
Black/African American	35 (16.4)
**Clinical characteristics**	
Adjudicated clinical diagnosis	
Normal cognition	146 (68.2)
Mild cognitive impairment	68 (31.8)
Adverse childhood experiences	
Emotional neglect	100 (46.7)
Family problems and separation	62 (29)
Parental intimidation	145 (67.8)
Parental violence	78 (36.4)
Financial need	45 (21)
Medical comorbidities	
Hypertension	79 (36.9)
Sleep apnea	36 (16.8)
Diabetes mellitus	17 (7.9)
Traumatic brain injury	3 (1.4)
Cardiovascular disease	12 (5.6)

Abbreviations: m, mean; SD, standard deviation.

### 3.2 ACEs

**FIGURE 1:**
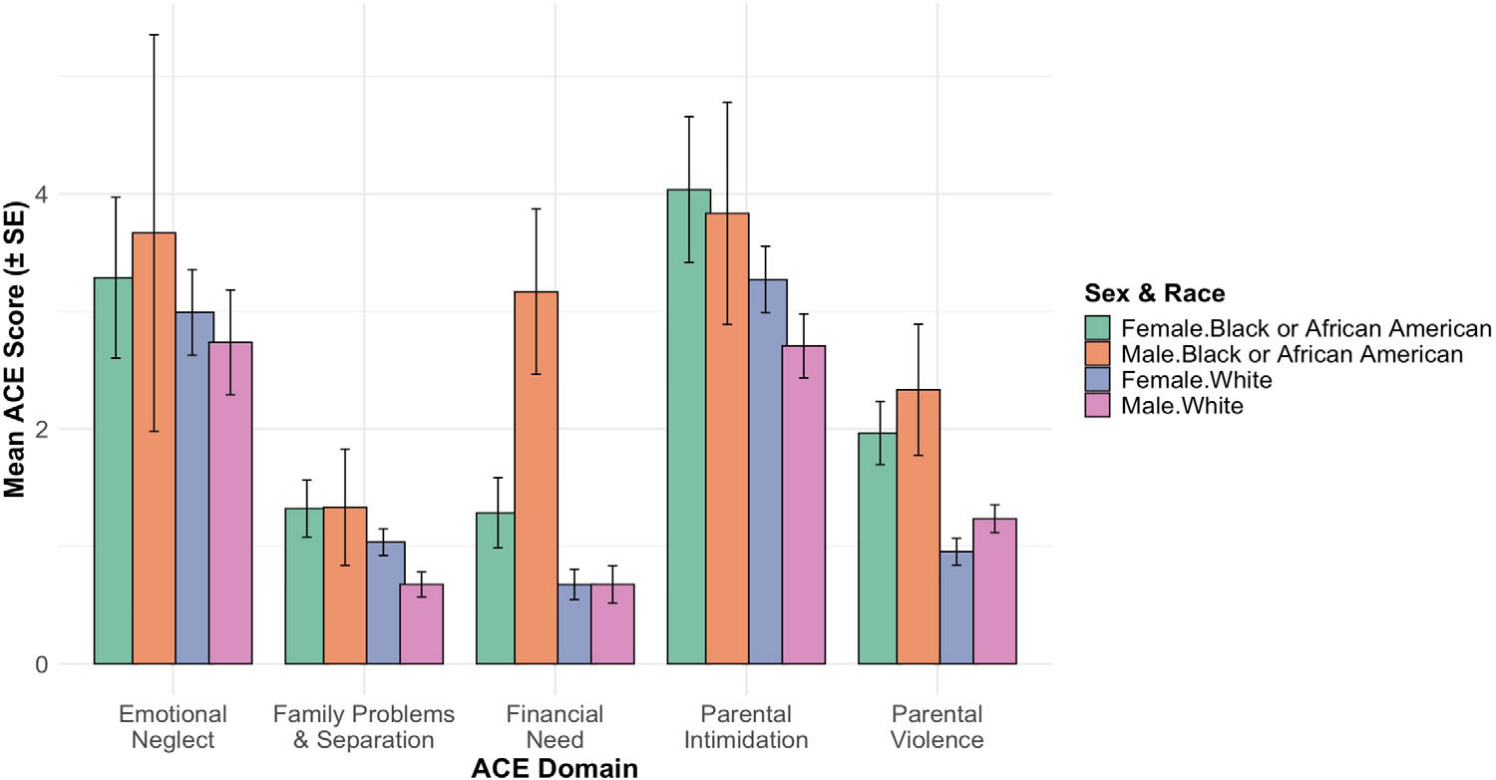
Mean scores across five adverse childhood experience (ACE) domains (emotional neglect, family problems & separation, financial need, parental intimidation, and parental violence) are shown by sex and race among older adults. Bar graphs represent average ACE domain scores with standard error (± SE) for each group: Black/African American women, Black/African American men, White women, and White men.

Parental intimidation was the most frequently reported ACE (67.8%), followed by emotional neglect (46.7%), parental violence (36.4%), family problems and separation (29%), and financial need (21%; Table 1). Family problems and separation were more frequently reported by females than males (*p* = 0.03). Financial need and parental violence significantly differed by race (*p *= 0.001 and *p* < 0.001, respectively; Figure 1), with Black participants reporting greater exposure to both (mean difference = 0.90, 95% confidence interval [CI]: –1.41 to –0.38 and mean difference = 0.94, 95% CI: –1.36 to –0.51, respectively).

### 3.3 Adjudicated clinical cognitive diagnoses and global cognition (MoCA scores)

**FIGURE 2:**
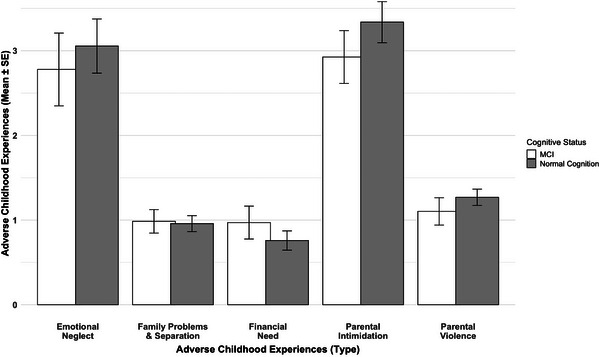
Bar graph showing average scores for five adverse childhood experience (ACE) domains among older Black and White adults with mild cognitive impairment (MCI) and those with normal cognition. SE, standard error

In linear regression models examining associations between clinical cognitive diagnoses and ACEs, no ACE domains demonstrated significant or trend‐level associations with adjudicated clinical diagnoses of NC (*n* = 146, 68.2%) or MCI (*n* = 68, 31.8%) in adjusted or unadjusted models (Figure 2). However, in linear regression models examining ACEs and their relationship with MoCA total scores, financial need was significantly associated with lower cognitive performance in both unadjusted (β = –0.10, 95% CI: –0.17 to –0.02, *p* = 0.01) and adjusted models (β = –0.07, 95% CI: –0.14 to –0.004, *p* = 0.04), while other ACE domains showed no significant associations with MoCA scores (Table S1 in supporting information). The Mann–Whitney *U* test revealed no significant differences in ACEs exposure between diagnostic groups (NC vs. MCI; all *p*  >  0.05). However, Spearman correlation demonstrated that greater financial need was significantly associated with lower MoCA scores (ρ = –0.19, *p* = 0.005).

### 3.4 Neuroimaging metrics

Higher parental violence scores were associated with a small but significant reduction in MD values (β = –0.007; 95% CI: –0.013 to –0.001; *p* = 0.029), suggesting an unexpected preservation of white matter integrity. While there were some trends between ACEs and other neuroimaging measures, these associations did not reach statistical significance in simple linear regression models. For example, parental violence trended toward higher NODDI values (*p* = 0.07), but after adjusting for demographic covariates, there were no significant associations between ACE domains and white matter neuroimaging metrics. All results are shown in Table S2 in supporting information.

### 3.5 Plasma biomarkers

**FIGURE 3:**
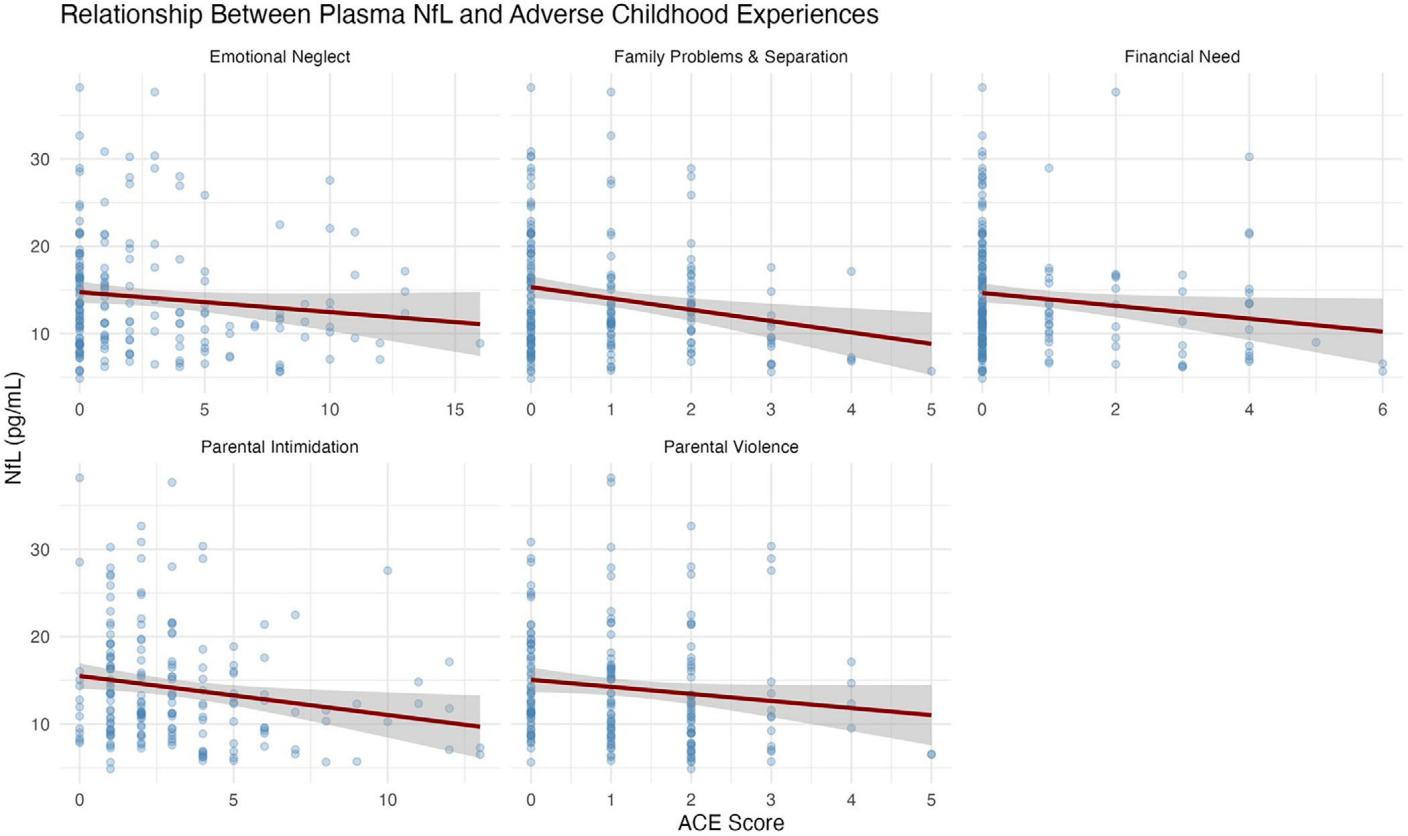
This figure presents the associations between plasma NfL concentrations (pg/mL) and scores across five adverse childhood experience (ACE) domains: emotional neglect, family problems & separation, financial need, parental intimidation, and parental violence. Each panel represents a separate linear regression model with scatterplots showing individual data points. Trend lines with 95% confidence intervals (shaded areas) illustrate the direction and strength of the associations across the full sample. NfL, neurofilament light chain.

**FIGURE 4:**
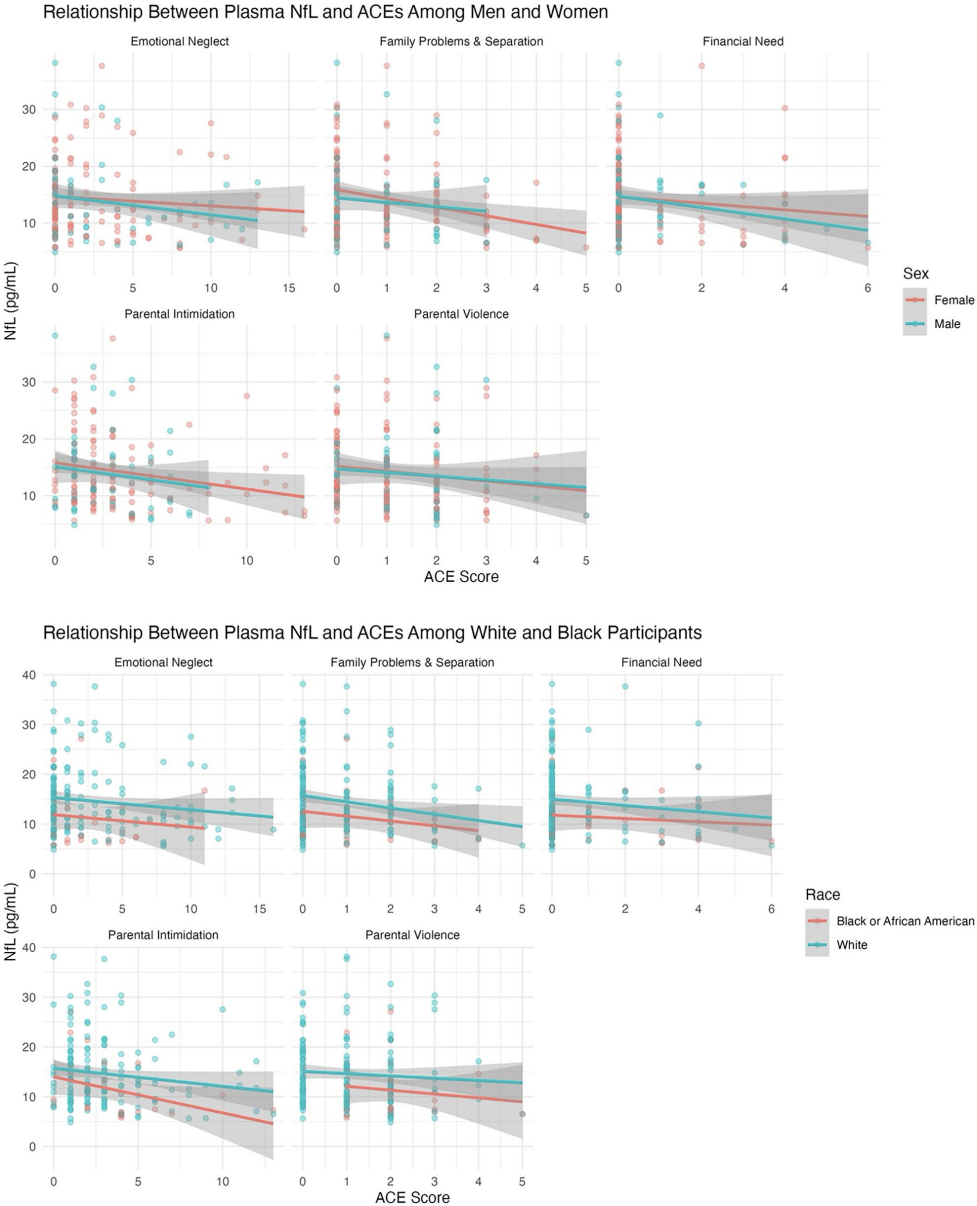
Scatterplots with fitted regression lines show associations between NfL concentrations (pg/mL) and individual ACE domains among Black/African American, White, men, and women participants. Each dot represents an individual ACE score; participants may have multiple dots corresponding to different ACE domains. ACE, adverse childhood experience; NfL, neurofilament light chain.

**FIGURE 5:**
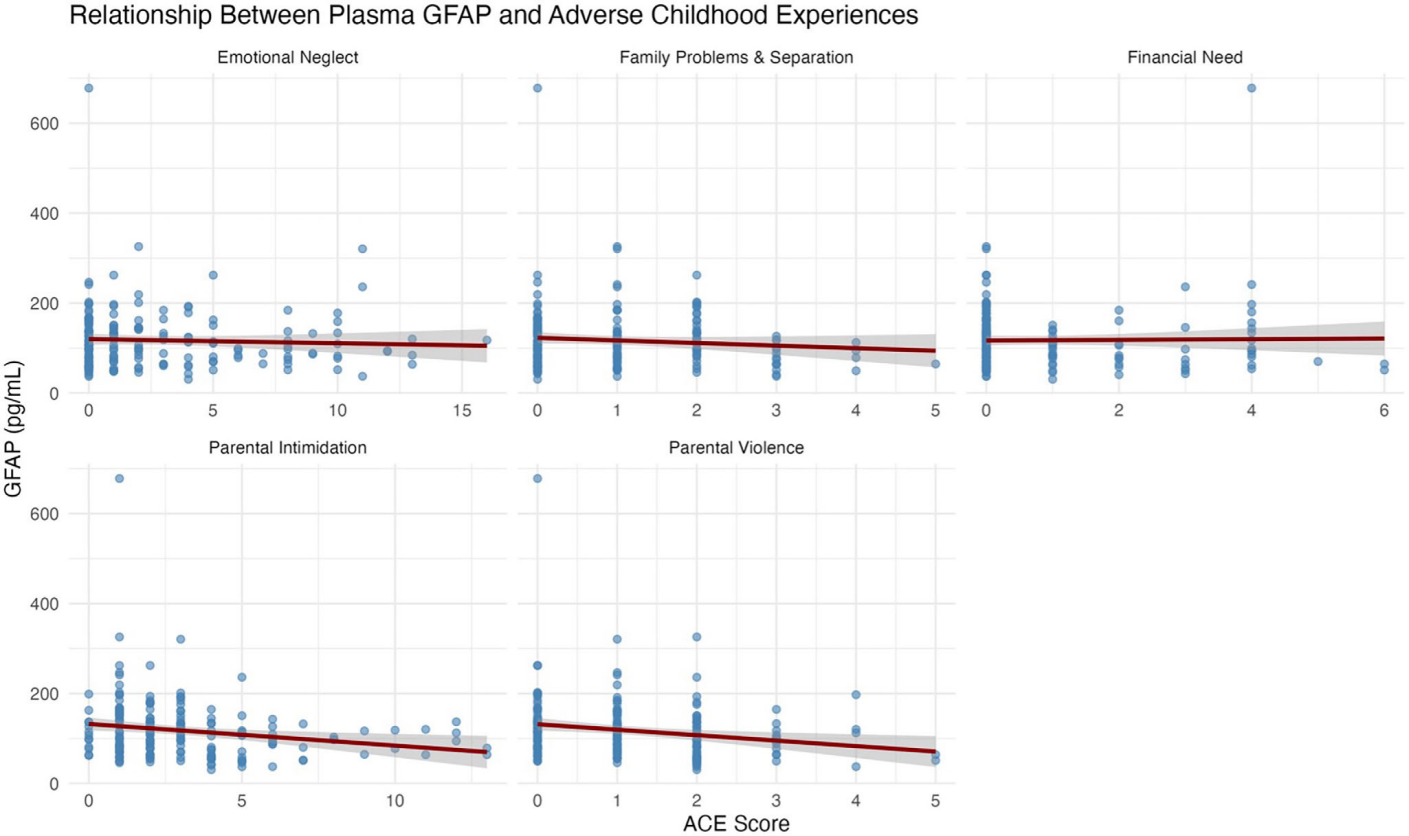
This figure displays the associations between plasma glial fibrillary acidic protein (GFAP) concentrations (pg/mL) and five adverse childhood experience (ACE) domains across the full study sample. Scatterplots show individual‐level data, and linear trend lines reflect unadjusted regression fits across ACE scores. Each panel represents a distinct ACE domain. One GFAP level of 678 pg/mL was not influential per diagnostic checks (Cook distance).

**FIGURE 6:**
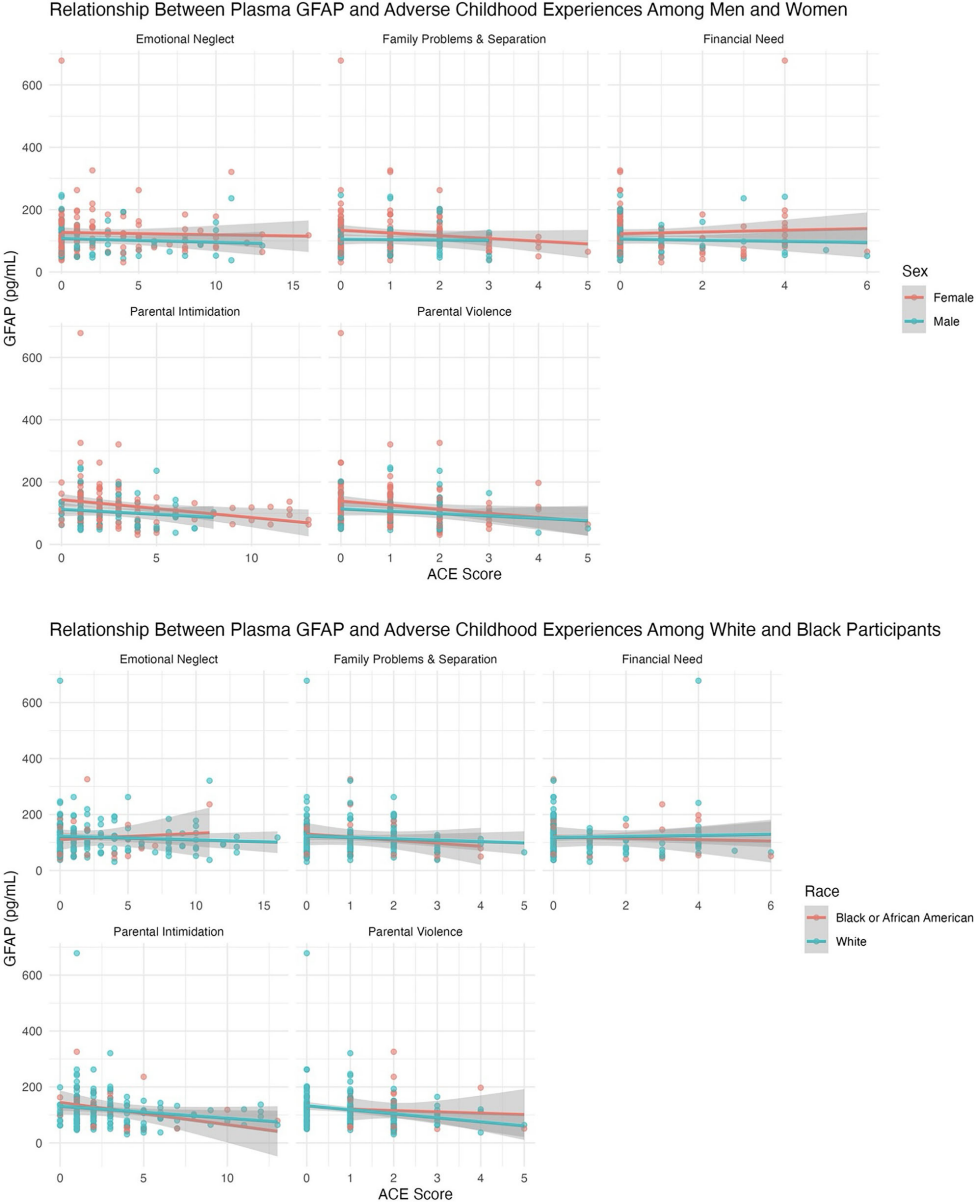
Stratified scatterplots with fitted linear regression lines depict the relationship between plasma glial fibrillary acidic protein (GFAP) concentrations (pg/mL) and five adverse childhood experience (ACE) domains among older adults. The top panels show associations by sex (female vs. male), and the bottom panels show associations by race (Black or African American vs. White). Each panel represents a separate ACE domain. Individual data points are plotted along with trend lines and 95% confidence intervals (shaded areas). These models adjust for age, education, and cognitive status (mild cognitive impairment vs. normal cognition).

There were several significant and borderline associations between all ACE domains and plasma biomarkers (NfL, GFAP, and Aβ42/40), while none were observed with p‐tau181. Prior to removing two influential NfL outliers (> 40 pg/mL), emotional neglect, family problems and separation, and parental intimidation were significantly negatively associated with NfL in both unadjusted and covariate‐adjusted models (all *p* <  0.05); financial need and parental violence were non‐significant (*p > *0.05). After outlier removal, all ACEs except emotional neglect and parental violence demonstrated consistent negative associations with NfL in unadjusted models (Table S3 in supporting information; Figure 3), though emotional neglect (*p *= 0.09) and parental violence (*p* =  0.07) did approach significance in unadjusted models. Only emotional neglect (β  =  –0.04; 95% CI: –‐0.07 to –0.01; *p*  =  0.01) and family problems and separation (β  =  –0.1; 95% CI: –0.2 to –0.004; *p*  =  0.04) showed an inverse association with NfL in covariate‐adjusted models (Figure 4). Parental intimidation and parental violence were negatively associated with GFAP levels in unadjusted models (*p *< 0.05; Figure 5). In adjusted models, only parental intimidation remained inversely related to GFAP levels (*p* < 0.05; Figure 6). Emotional neglect demonstrated a borderline negative association with Aβ42/40 (*p*  =  0.06) after covariate adjustment.

## 4 DISCUSSION

This study sought to understand how ACEs relate to fluid and neuroimaging ADRD biomarkers and cognition in a cohort of Black and White older adults. While early‐life adversity contributes to dysregulation of the HPA axis, chronic inflammation, and poor health outcomes in adulthood,^17^ the specific domains of ACEs and their relationships to fluid and neuroimaging ADRD biomarkers and cognitive impairment were previously underexplored. Our findings present a complex picture, with multiple ACE domains showing associations that contrast expectations based on prior literature.

One major unexpected result of this study was a significant association between parental violence and MD in white matter tracts. Higher levels of reported parental violence were associated with lower MD values, which typically suggest better white matter integrity. This finding is unexpected, as ACEs have been associated with poor white matter health.^41^ One possible explanation is that lower MD values may reflect altered tissue microstructure rather than true preservation of white matter integrity. Future research incorporating additional neuroimaging modalities, such as radial and axial diffusivity, diffusion kurtosis imaging, or free water imaging, may clarify whether this association represents a neurobiological resilience mechanism or a compensatory adaptation to early adversity. Notably, parental violence also trended toward higher NODDI values, which suggests greater neurite density. This trend is consistent with typical interpretations of NODDI in the context of white matter health, in which elevated values may reflect white matter abnormalities and degeneration. Overall, however, these findings should be interpreted with caution given the limited sample size, and future longitudinal studies are needed to clarify the biological significance of this relationship.

This study revealed several unexpected relationships between certain ACEs and plasma biomarkers of neurodegeneration. For example, family problems and separation, financial need, and parental intimidation were significantly associated with lower NfL levels, and both parental violence and parental intimidation were significantly associated with lower GFAP levels. These findings contrast with prevailing literature, which often associates ACEs with elevated markers of inflammation and neurodegeneration.^42^, ^43^ In addition, elevated NfL levels are typically indicative of increased neuronal damage and have been linked to neurodegenerative processes, neuroinflammation, and disruptions in brain structural integrity.^44^, ^45^ Thus, the literature posits that these early life experiences would contribute to long‐term neurobiological vulnerability, potentially accelerating axonal degeneration in aging individuals.^12^, ^46^

This insight expands current knowledge by demonstrating the potential for diverse ACE domains to differentially influence biological pathways underlying neurodegeneration and cognitive health, particularly in the context of ADRD. These findings of lower biomarker levels in association with higher ACEs may indicate alternative mechanisms, including early neuronal loss, downregulation of inflammatory pathways, or compensatory neuroprotective responses that emerge in the context of chronic stress. For instance, chronic exposure to early trauma may initially heighten inflammatory processes but later result in a regulatory feedback loop that suppresses inflammation as a survival adaptation. These findings align with prior work demonstrating HPA axis dysregulation in populations exposed to early trauma.^17^ Dysregulated cortisol dynamics, a hallmark of HPA axis dysfunction, may contribute to altered neuroinflammatory and neurodegenerative pathways.^47^

A key strength of this study is its comprehensive approach to examining ACEs in the context of both plasma biomarkers and neuroimaging metrics, offering a multidimensional view of the long‐term impacts of childhood adversity on brain health. However, several key study design and conceptual considerations must be acknowledged. First, the cross‐sectional nature of the study precludes causal inferences about the relationship between ACEs and neurodegeneration. Second, the reliance on self‐reported ACEs introduces potential recall bias, particularly in older adults with MCI, as individuals with cognitive impairment may have differential memory for past adversity, possibly underreporting or overreporting experiences based on current psychological state or cognitive function. Third, survival bias may have influenced the findings, as individuals who experienced more severe outcomes related to ACEs may not be well represented in the cohort, leading to an overrepresentation of individuals who were either resilient to early adversity or who developed adaptive coping mechanisms that mitigated its long‐term impact. The study's relatively high education levels further limit generalizability, as participants with higher socioeconomic status may be more resilient to the long‐term effects of early‐life adversity. Finally, the lack of significant findings in neuroimaging metrics may be attributable to the relatively small sample size and absence of longitudinal imaging data. Future research should address these limitations through longitudinal studies that track changes in cognitive diagnosis, domain‐specific performance, and neuroimaging markers in diverse cohorts. Moreover, investigating the molecular mechanisms underlying the observed associations, such as the role of inflammatory markers or stress‐related neurohormones, could provide deeper insights into the pathways linking ACEs to ADRD risk. Proteomic and metabolomic approaches may also be particularly valuable to identify novel biomarkers and therapeutic targets. Further, stratified analyses by apolipoprotein E ε4 carrier status or other genetic risk factors could clarify the interplay between genetic predisposition and early life adversity.

Significant differences were observed by race in reports of parental violence and financial need. However, no racial differences were found across other ACE domains or in clinical diagnosis, plasma biomarkers of AD and neurodegeneration, or neuroimaging measures of white matter integrity. Future research on ACEs that incorporates examination of race‐based differences should move beyond racial categories and incorporate more direct, theory‐driven measures of social and structural adversity, such as perceived discrimination, neighborhood disadvantage, and barriers to health‐care access, as these factors underlie health disparities.^48^, ^49^, ^50^, ^51^ In addition, given the overall limited racial and ethnic representation in this cohort, future studies should include larger and more diverse samples to improve generalizability and ensure that historically marginalized populations are adequately represented in aging and dementia research.

Financial need showed a significant association with lower MoCA scores, which is consistent with research linking socioeconomic adversity to reduced cognitive performance in later life. There is well‐documented literature on the long‐term impact of poverty and material deprivation on cognitive development and educational attainment. Individuals exposed to financial hardship in childhood may experience persistent stress, reduced access to cognitively enriching environments, and lower education quality, all of which can have cumulative effects on cognitive reserve.

As for neuroimaging, future work should leverage additional neuroimaging measures available in this cohort to clarify interpretation of the MD and WMH findings. While this study focused on white matter structural integrity, future analyses incorporating complementary metrics such as volumetric assessments from FreeSurfer and cerebral blood flow measurements could provide deeper insights into the mechanisms linking ACEs to neurodegeneration. These measures would allow for more nuanced evaluation of whether lower MD values associated with parental violence reflect true preservation of white matter integrity or potential differences in tissue microstructure that are not captured by DTI alone. Further, longitudinal studies tracking changes in white matter integrity over time could help disentangle the role of ACEs in neurodevelopmental versus neurodegenerative processes, addressing whether these observed relationships persist, worsen, or shift with aging.

In conclusion, this study highlights the complex and nuanced relationships between early‐life adversity and markers of neurodegeneration in a cohort of Black and White older adults. While ACEs are often linked to adverse health outcomes, our findings showed unexpected patterns, including significant associations between specific ACE domains and lower levels of plasma neurodegeneration biomarkers, as well as an association between parental violence and lower MD values, suggesting preserved white matter integrity. These results challenge conventional assumptions about the detrimental effects of early‐life adversity on neurobiological aging and highlight the need for further research to clarify potential compensatory or resilience mechanisms.

## CONFLICT OF INTEREST STATEMENT

None of the authors have conflicts of interest related to these data. Author disclosures are available in the supporting information.

## Supporting information



Supporting Information

Supporting Information
