# Supplementary material for: Adverse childhood experiences influence markers of neurodegeneration risk in older adults
Source: Alzheimers Dement. 2025 Jul 30;21(8):e70523. doi: 10.1002/alz.70523 (PMC12310557; doi:10.1002/alz.70523)
Supplement: Supplementary file 1 — Supporting Information [file ALZ-21-e70523-s001.docx]

**Supplemental Methods: Diffusion MRI Acquisition and Processing**

MRI Acquisition Parameters

All imaging data were collected using a research-dedicated 3T Siemens Skyra scanner equipped with a 32-channel head coil at Wake Forest School of Medicine. The diffusion-weighted imaging (DWI) protocol included a multi-shell acquisition with 99 diffusion directions across multiple b-values (b = 0, 1000, 2000 s/mm²), enabling both DTI and NODDI model estimation. Voxel size was 2.0 mm³ isotropic, with a field of view (FOV) of 256 mm and TR/TE = 3000/89 ms. T1-weighted anatomical images were also collected (MPRAGE sequence; 1.0 mm³ isotropic voxels) to support co-registration and normalization.

DTI Processing (Fractional Anisotropy and Mean Diffusivity)

Diffusion data were preprocessed using the FMRIB Software Library (FSL), including correction for eddy currents and head motion using the eddy tool, and brain extraction using BET. The diffusion tensor was modeled using Camino, and voxelwise maps of fractional anisotropy (FA) and mean diffusivity (MD) were generated. These maps were then co-registered to the individual’s T1-weighted image, followed by normalization to MNI space via ANTs nonlinear registration. Prior to region-of-interest (ROI) analysis, all FA and MD maps were visually inspected for quality assurance.

NODDI Processing (Intracellular Volume Fraction)

NODDI model fitting was conducted using the Accelerated Microstructure Imaging via Convex Optimization (AMICO) framework, which estimates three microstructural indices: intracellular volume fraction (ICVF), isotropic volume fraction (ISO), and orientation dispersion index (ODI). For this analysis, only ICVF was used. NODDI maps were aligned to MNI space following the same pipeline used for DTI maps (T1 co-registration and ANTs normalization).

ROI Extraction and White Matter Summary Metrics

To obtain global white matter summary values for FA, MD, and ICVF, we applied the Johns Hopkins University (JHU) ICBM-DTI-81 white matter atlas in MNI space. Values were extracted from all supratentorial white matter ROIs and averaged to compute tract-weighted means. Prior to averaging, mean diffusivity (MD) values were normalized across participants to account for inter-individual variation in scale and distribution. FA and ICVF values were not further transformed, as these are inherently normalized and bounded (0–1).

**SUPPLEMENTAL TABLES**

| **Predictor ACE** | **Outcome** | **ß-coefficient** | **Lower CI** | **Upper CI** | ***P*** |
| --- | --- | --- | --- | --- | --- |
| Emotional neglect | Global Cognition (MoCA Total Score) | 0.0061 | -0.0231 | 0.0353 | 0.6807 |
|  |  |  |  |  |  |
| Family problems & separation |  | -0.0055 | -0.1023 | 0.0912 | 0.9104 |
|  |  |  |  |  |  |
| Financial need |  | -0.0964 | -0.1708 | -0.022 | 0.0113* |
|  |  |  |  |  |  |
| Parental intimidation |  | 0.0186 | -0.0202 | 0.0574 | 0.3447 |
|  |  |  |  |  |  |
| Parental violence |  | -0.0213 | -0.1108 | 0.0682 | 0.6392 |

Supplemental Table 1. ACEs and Montreal Cognitive Assessment (MoCA) Scores

Linear regression models examined associations between individual adverse childhood experience (ACE) domains and global cognitive function as measured by total MoCA score. β-coefficients represent the estimated change in MoCA total score per unit increase in each ACE domain. All models are unadjusted. Asterisks (*) indicate statistically significant associations at p < 0.05.

| **Predictor ACE** | **Outcome** | **ß-coefficient** | **Lower CI** | **Upper CI** | ***P*** |
| --- | --- | --- | --- | --- | --- |
| Emotional neglect | DTI fractional anisotropy | -7E-04 | -0.0016 | 2E-04 | 0.1253 |
|  | Mean diffusivity | 4E-04 | -0.0015 | 0.0023 | 0.703 |
|  | NODDI intracellular volume fraction | -2E-04 | -0.0019 | 0.0014 | 0.8024 |
| Family problems & separation | DTI fractional anisotropy | 0.001 | -0.002 | 0.0041 | 0.5021 |
|  | Mean diffusivity | -0.0033 | -0.0096 | 0.003 | 0.298 |
|  | NODDI intracellular volume fraction | 6E-04 | -0.0049 | 0.0061 | 0.8231 |
| Financial need | DTI fractional anisotropy | 3E-04 | -0.0022 | 0.0028 | 0.8019 |
|  | Mean diffusivity | -0.0039 | -0.009 | 0.0012 | 0.1312 |
|  | NODDI intracellular volume fraction | 0.0028 | -0.0017 | 0.0072 | 0.2172 |
| Parental intimidation | DTI fractional anisotropy | 2E-04 | -0.0011 | 0.0014 | 0.7876 |
|  | Mean diffusivity | -0.0014 | -0.0039 | 0.0012 | 0.2836 |
|  | NODDI intracellular volume fraction | 9E-04 | -0.0013 | 0.0032 | 0.4099 |
| Parental violence | DTI fractional anisotropy | 0.0014 | -0.0016 | 0.0044 | 0.3653 |
|  | Mean diffusivity | -0.0069 | -0.0131 | -7E-04 | 0.0294* |
|  | NODDI intracellular volume fraction | 0.0048 | -6E-04 | 0.0102 | 0.0782 |

Supplemental Table 2. ACEs and white matter neuroimaging measures

Linear regression models examined links between each adverse childhood experience (ACE) domain and white matter neuroimaging measures: fractional anisotropy, mean diffusivity, and neurite orientation dispersion and density imaging (NODDI) intracellular volume fraction. β-coefficients reflect standardized effects of ACEs on unadjusted neuroimaging metrics, averaged across white matter tracts defined by the JHU atlas.

| **Predictor ACE** | **Outcome** | **ß-coefficient** | **Lower CI** | **Upper CI** | ***P*** |
| --- | --- | --- | --- | --- | --- |
| Emotional neglect | P-tau 181 | 0.0023 | -0.0382 | 0.0428 | 0.9117 |
|  | NFL | -0.0385 | -0.0786 | 0.0016 | 0.0596 |
|  | GFAP | -0.0138 | -0.0542 | 0.0267 | 0.503 |
|  | Aß42/40 | 0.0266 | -0.0137 | 0.0669 | 0.194 |
| Family problems & separation | P-tau 181 | -0.0838 | -0.2144 | 0.0468 | 0.2071 |
|  | NFL | -0.2061 | -0.3338 | -0.0785 | 0.0017* |
|  | GFAP | -0.086 | -0.2166 | 0.0446 | 0.1953 |
|  | Aß42/40 | -0.0184 | -0.1495 | 0.1128 | 0.7828 |
| Financial need | P-tau 181 | 0.0127 | -0.0916 | 0.117 | 0.8104 |
|  | NFL | -0.0609 | -0.1648 | 0.0429 | 0.2485 |
|  | GFAP | 0.0123 | -0.0919 | 0.1165 | 0.8159 |
|  | Aß42/40 | -0.041 | -0.1451 | 0.0631 | 0.4379 |
| Parental intimidation | P-tau 181 | 7E-04 | -0.0528 | 0.0541 | 0.9804 |
|  | NFL | -0.0715 | -0.1238 | -0.0191 | 0.0077* |
|  | GFAP | -0.0716 | -0.124 | -0.0193 | 0.0076* |
|  | Aß42/40 | 0.0366 | -0.0166 | 0.0897 | 0.176 |
| Parental violence | P-tau 181 | 0.0091 | -0.1228 | 0.141 | 0.8921 |
|  | NFL | -0.1352 | -0.2654 | -0.0051 | 0.0418* |
|  | GFAP | -0.1783 | -0.3074 | -0.0492 | 0.007* |
|  | Aß42/40 | 0.0531 | -0.0784 | 0.1845 | 0.4267 |

Supplemental Table 3. ACEs and biomarkers

Associations between ACE domains and plasma biomarkers. Linear regression models tested associations between ACE domains and standardized concentrations of p-tau181, NfL, GFAP, and amyloid-β42/40. All biomarkers were Z-score standardized prior to analysis.
